# Solubilization of Hydrophobic Dyes in Surfactant Solutions 

**DOI:** 10.3390/ma6020580

**Published:** 2013-02-21

**Authors:** Ali Reza Tehrani-Bagha, Krister Holmberg

**Affiliations:** 1 Department of Chemical and Biological Engineering, Chalmers University of Technology, Göteborg SE-412 96, Sweden; E-Mail: tehrani@chalmers.se; 2 Institute for Color Science and Technology, Tehran 16765-654, Iran

**Keywords:** dye, water insoluble, hydrophobic, surfactant, solubilization, micelle

## Abstract

In this paper, the use of surfactants for solubilization of hydrophobic organic dyes (mainly solvent and disperse dyes) has been reviewed. The effect of parameters such as the chemical structures of the surfactant and the dye, addition of salt and of polyelectrolytes, pH, and temperature on dye solubilization has been discussed. Surfactant self-assemble into micelles in aqueous solution and below the concentration where this occurs—the critical micelle concentration (CMC)—there is no solubilization. Above the CMC, the amount of solubilized dye increases linearly with the increase in surfactant concentration. It is demonstrated that different surfactants work best for different dyes. In general, nonionic surfactants have higher solubilization power than anionic and cationic surfactants. It is likely that the reason for the good performance of nonionic surfactants is that they allow dyes to be accommodated not only in the inner, hydrocarbon part of the micelle but also in the headgroup shell. It is demonstrated that the location of a dye in a surfactant micelle can be assessed from the absorption spectrum of the dye-containing micellar solution.

## 1. Introduction

The use of surfactants for solubilization of dyes dates back to the 1940s [1,2,3,4,5,6]. For the majority of applications an aqueous medium is used and the choice of surfactant is decisive of the adsorption and fixation of the dye at the substrate (e.g., textile fiber/fabric) [7]. Moreover, the surfactant is essential for removing poorly bound dye from the substrate by a solubilization mechanism in an after-treatment washing procedure. This paper reviews the use of surfactants for solubilization of hydrophobic dyes.

Surfactants are also used to create suspensions and emulsions of dyes. Such systems differ from the solubilized systems in several respects. A fundamentally important difference is that whereas solubilized systems are thermodynamically stable, dispersions such as suspensions and emulsions are never stable in a thermodynamical sense; they will eventually undergo phase separation [8,9]. Dispersed systems will not be dealt with in this review.

Another limitation of this review is that it deals mainly with hydrophobic dyes. Water soluble dyes do not need surfactants to go into aqueous solution. However, surfactants may still influence the binding of such dyes to the substrate and this topic has been reviewed elsewhere [10,11,12,13].

Other hydrophobic substances may also be solubilized by surfactants and the general concept is the same regardless of type of solubilisate. Solubilization of hydrophobic drug molecules has been a particularly active area and several reviews can be found on the topic [14,15,16,17,18]. Solubilization of hydrophobic dyes seems not to have been reviewed before, however.

## 2. Dyes

Dyes are unsaturated organic substances that absorb part of the visible light. They should have an affinity to substrates such as textiles, paper, *etc*. The introduction of synthetic dyes from petroleum sources in the late 19th century ended the market for natural dyes from plant origin, which had been in use since 3500 BC. Today, more than 100,000 different dye structures have been synthesized and it is estimated that more than 3600 individual dyes are being produced for commercial purposes [19,20]. The world consumption of organic dyes is linked to the global fiber production [21]. The current world-wide production of fiber is around 80 million tons per year [22,23] and the total dye consumption is over 1 million tons.

The organic dyes can be classified according to their chemical structure into groups such as azo compounds (60%–70%), anthraquinones (15%), triarylmethanes (3%), phthalocyanines (2%), *etc*., with the values within parenthesis indicating per cent of total volume. They may also be classified according to application: acid dyes (16%), disperse dyes (18%), direct dyes (16%), reactive dyes (13%), *etc*. More detailed information about the classification and about the applications for the different classes can be found in [7,24]. One class of dye may be used for different applications.

This paper deals with solubilization of hydrophobic dyes. To make the picture more complete, we also briefly mention that there exist water soluble dyes as well. These need not be solubilized by surfactants when used in aqueous solution. The water soluble dyes carry a charged group, which may be anionic or cationic. Anionic dyes normally have a sulfonate or a carboxylate substituent. A sulfonic acid is very acidic, which means that a dye with a sulfonate group remains anionic over the entire pH range. A carboxylic acid, on the other hand, is a weak acid and a relatively high pH is needed for a carboxylate-containing dye to be anionic and water soluble. Anionic dyes are suitable for coloration of wool, silk and certain polyamide fibers. The dyeing process is often made in acidic solution, where these fibers are positively charged through protonation of amino groups. Dyes carrying sulfonate groups are then preferred because they will remain anionic also at low pH, as mentioned above.

**Figure 1:**
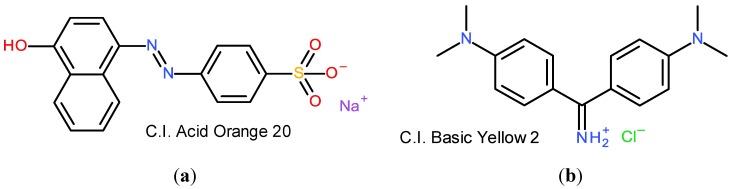
(**a**) An anionic azo dye with a sulfonate substituent; (**b**) and a cationic dye with a diphenylmethane structure and a quaternary ammonium group.

Cationic dyes, or basic dyes, have one or more quaternary ammonium substituents. They are used almost exclusively for coloration of acrylic fibers, *i.e.*, fibers based on acrylonitrile as the main monomer. These fibers are negatively charged, which means that they interact strongly with a positively charged dye molecule. Figure 1 shows one example of an anionic dye and one of a cationic dye. As discussed above, the charged functional groups in the structures serve two purposes: (1) they render the dye water soluble; and (2) they provide anchoring of the dye to oppositely charged fiber surfaces.

Dyes that are insoluble or poorly soluble in water are divided into (a) disperse dyes that are normally used for coloration of plastics and synthetic fibers; (b) solvent dyes for coloration of solvents, gasoline, ink, fats, oils, *etc*.; and (c) sulfur and vat dyes for coloration of cotton fibers. A commercial dyeing formulation may consist of a complex mixture of ingredients. These are usually proprietary mixtures and seldom revealed in the open literature. A patent example of ingredients in such a formulation is provided in Table 1.

**Table 1:** Ingredients used in a dyestuff formulation [25].

Purpose	Chemicals
Dispersants/Surfactants	lignin sulfonates, naphthalene sulfonate, formaldehyde condensates, ethylene oxide-propylene oxide block copolymers
Salts	sodium sulfate, sodium chloride
Dust-bonding agents	mineral oils, paraffin oils (+additives)
Antifoams	ditertiary acetylene glycols
Antifreezes	glycerol, glycols
Thickeners	carboxymethylcellulose, polyacrylates
Buffer systems	phosphate, acetate

## 3. Coloration with Hydrophobic Dyes

### 3.1. Coloration with Disperse and Solvent Dyes

Disperse dyes, with around 20% of the total dye consumption [7], were originally developed for coloration of cellulose acetate fibers in the early 1920s [26]. Nowadays, they are used mainly for dyeing of polyester fibers and to lesser extent for other hydrophobic fibers such as polyamide (Nylon), and polyacrylonitrile (Acrylic) [27]. Disperse dyes have sparingly low solubility in water but they are soluble in the polymeric fiber at elevated temperatures.

Before use, the disperse dye is finely ground in the presence of a dispersing agent. The dye-dispersant mixture is then sold as a paste or spray or powder. The very fine particle size gives a large surface area, which is beneficial for the dissolution process. The dispersed dye binds to polar hydrophobic fibers by a combination of intermolecular interactions, e.g., hydrogen bonding, and van der Waals attraction forces [21].

Disperse dyes are typically small, planar and non-ionic molecules with polar substituents such as -NO_2_ and -CN. The planar geometry allows the dye to penetrate in-between tightly packed polymer chains, and the polar groups are essential for the interaction with the polymer, *i.e.*, for the retention of the dye to the fiber surface. The polar group also influences the UV adsorption of the molecule, which in turn, affects the color of the dye. The dye is generally applied in an aqueous solution under pressure and at high temperatures (120–130 °C). At this temperature range, thermal agitation causes the polymer's structure to become looser and less crystalline, opening gaps for the dye molecules to enter [21,28,29,30].

A disperse dye in an aqueous dyeing process needs to be solubilized by the help of a surfactant. For such systems the surface active agent is a vital component of the formulation and the dyeing efficiency is governed by the choice of surfactant. The use of surfactant for solubilizing such dyes is the central theme of this chapter but before entering into this topic the basics for coloration with the two types of hydrophobic dyes that do not need to be solubilized by surfactants, the vat and the sulfur dyes, will be briefly described.

### 3.2. Coloration with Vat and Sulfur Dyes

Vat and sulfur dyes, with around 20% of the total dye consumption, are two other classes of dyes that are not soluble in water. Their solubility may be increased by chemical modification, an example of which is given below.

**Figure 2:**
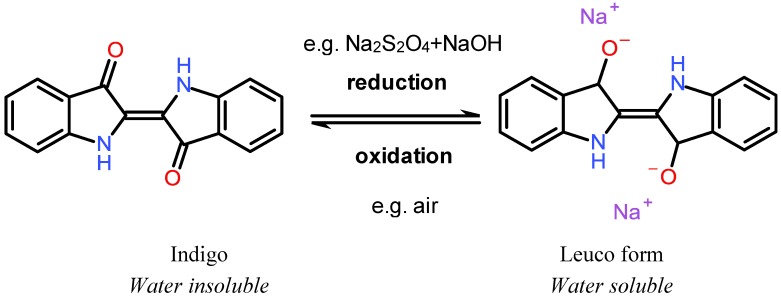
The intensely colored and hydrophobic indigo dye can be reduced to water soluble leuco form. The leuco form is easily oxidized back to indigo.

Indigo, the common blue dye of blue jeans, is one of the traditional and the most important member of vat dyes family. It used to be extracted from plants and used for textile coloration in India, China, and Egypt since 2000 B.C. Synthetic indigo was developed by the German chemist von Baeyer at the end of the nineteenth century and it rapidly took over the market from natural indigo dye [31]. The original indigo is not soluble in water but it can readily be made water soluble by thiosulfate reduction to the so-called leuco form, “white indigo” (see Figure 2). The leuco indigo is a at high pH and consequently soluble in alkali. In its ionized form, it can be applied to the cotton fiber. Once attached to the fiber, the leuco indigo quickly combines with oxygen in the air and reverts to the insoluble, intensely colored dye.

Textile coloration is by far the most important application of vat dyes but a wide variety of other applications have also emerged, often requiring dyes with specific chemical structures. Examples of such applications are imaging, displays, memory technologies, analytical chemistry, indicators for biological sciences, *etc*. [32]. Some of the vat dyes have been applied to hydrophobic fibers and their blends with cotton. It is an advantage if such blend fibers can be treated in a single dye system [33].

**Figure 3:**
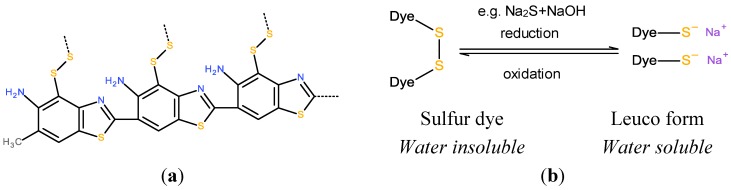
(**a**) an example of a sulfur dye [37]; (**b**) The intensely colored and hydrophobic sulfur dye can be reduced to the water soluble leuco form. The leuco form is oxidized back to the colored form.

As the name implies, sulfur dyes are sulfur containing unsaturated molecules. They are often used for dark colors, such as black and brown. They are often complex mixtures and the precise chemical structure is sometimes not known. Sulfur dyes are water insoluble and similar to the vat dyes they are usually used in the leuco form in the coloration process. Sodium sulfide is a common reducing agent, splitting disulfide bridges into thiol groups. Thiols have a pKa in the range 10–11, which means that the reduced molecule becomes ionized and water soluble in moderately strong alkali. In this form the dye adsorbs on cotton (Figure 3) [34,35,36].

In the examples of vat and sulfur dyes given above, the dyes have been converted into a water soluble form by a mild reduction procedure. This water soluble form, which is anionic, interacts with the fiber surface. Once it has diffused into the substrate, the colored and water insoluble form of the dye is recreated by oxidation. Therefore, these dyes do not normally desorb and bleed from the dyed fabric during the washing process, *i.e.*, they have high washing fastness.

## 4. Surfactants

Surfactants, surface active agents, are amphiphilic organic compounds with a strong tendency to adsorb at surfaces and interfaces. They consist of a hydrophobic tail and a hydrophilic head group [9]. The hydrophobic tail can be branched or linear; aliphatic, alkylaryl; short or long (typically between 8 and 18 carbon atoms in a straight alkyl chain). The hydrophilic head group may be ionic or non-ionic. Thus, the chemical structure of surfactants can vary widely [8].

Surfactants are present in many products that we use in our daily life such as soaps, detergents, shampoos, shower gels, creams, cosmetics, foods, drugs, *etc*. [8,38]. The global production of surfactants was estimated to 12 M tons/year in 2008 with a growth rate of 2%–3% per year [39,40]. 

Surfactant unimers in aqueous solution self-assemble into micelles at a specific concentration called the critical micelle concentration, or the CMC. Above the CMC, the unimer concentration remains the same; the additional surfactants just form more and more micelles. The CMC is an important characteristic, specific to each individual surfactant. Surfactant micelles are dynamic entities and can have different shapes, such as spherical, spheroid, oblate and prolate. Since the shape can vary, so does the size of the micelle.

**Figure 4:**
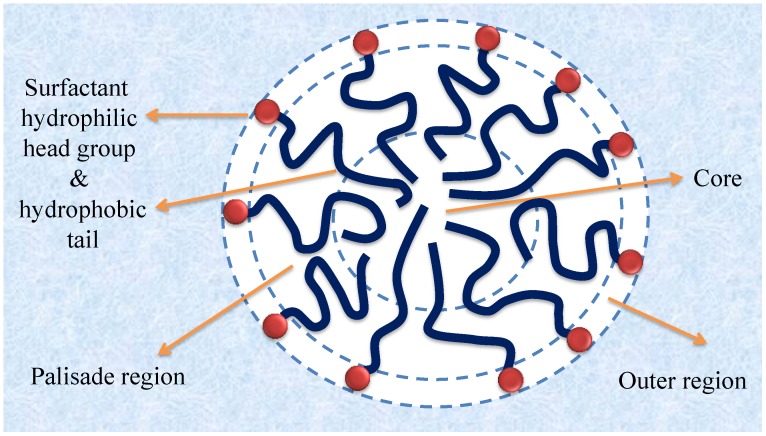
A surfactant micelle in water. Three different regions can be identified: the outer region, the palisade region and the core.

Figure 4 shows a schematic illustration of a spherical micelle. A surfactant micelle can be subdivided into three regions: (a) the interface between the micelle and the surrounding bulk water, also called the outer region, for ionic surfactants known as the Stern layer, where the hydrophilic head groups of the surfactants are present; (b) the outer part of the hydrophobic tail region, sometimes referred to as the palisade region; and (c) the core of the micelle. The latter region constitutes a very hydrophobic pseudophase and there is a gradient of increasing polarity from the core toward the micelle surface [9]. The average number of surfactants in each micelle and the shape of the micelles can be studied by a number of experimental techniques, such as small-angle neutron scattering (SANS), small-angle X-ray scattering (SAXS), cryo-transition electron microscopy (Cryo-TEM), steady-state fluorescence quenching (SSFQ), time-resolved fluorescence quenching (TRFQ), dynamic light scattering (DLS) and pulsed gradient spin-echo NMR (PGSE NMR). Each method gives its own unique contribution to the elucidation of the structure [41].

The shape and size of the micelles can influence various properties of a surfactant solution, such as viscosity and solubilization. The most important factor that determines the shape of the micelle is the geometry of the surfactant. The critical packing parameter (CPP) which gives a geometric characterization of a surfactant molecule is a useful number to predict the aggregate structure Equation 1:

CPP = ν/(l_max_.a_0_) (1)

In Equation 1, ν is the volume of the hydrophobic alkyl tail; l_max_ the extended length of the alkyl chain; and a_0_ the cross sectional area occupied by the polar head group at the micelle-solution interface. As a general rule, CPP values below 1/3 give spherical micelles, values between 1/3 and 1/2 give rod-like micelles and when the CPP values approach unity vesicles are formed instead of micelles [9].

## 5. Standard Test Method for Dye Solubilization

For measuring the solubilization of a water-insoluble dye in water, a calibration curve of the dye in a mixture of water and a good solvent (e.g., 50% v/v ethanol or acetone) is first constructed across a suitable range of dye concentrations. UV-Vis spectroscopy is the most commonly used technique for quantitative characterization of the dye [42,43,44,45]. The absorbance values at the wavelength of maximum absorption (λ_max._) *vs*. dye concentration is linear and can be used for determination of the dye molar extinction coefficient (ε) using the Beer-Lambert law (Equation 2):

(2) A = εlc

where A is absorbance; l the light path length; and c the dye concentration. It should be noted that this law has its own limitations and there is a deviation from a linear relation between A and c at high dye concentrations [46].

Solubilization of the dye in aqueous surfactant solutions is then investigated. The surfactant concentration should be varied from below to several times above the CMC. The normal procedure is to add an excess of finely powdered dye to the surfactant solution, keep the suspension under stirring until equilibrium is reached (normally 24–48 h at room temperature) and then remove the non-solubilized dye by filtration or centrifugation. The filtrate is subsequently diluted with an equal volume of the solvent that was used for preparation of the calibration curve. The absorption of the solution is recorded in a UV-Vis spectrophotometer and the concentration of solubilized dye is determined from the calibration curve (*i.e.*, from Equation 2 by knowing the ε at λ_max_).

The molar solubilization capacity or solubilization power (SP) of a surfactant is defined as moles of solubilized dye per mole of micellized surfactant (Equation 3):

(3) $$\mathrm{SP}\;=\;\frac{S_{\mathrm{total}}-S_{\mathrm{wat}}}{C_{\mathrm{surf}.}-\mathrm{CMC}}$$

where S_total_ is the molar solubility of the dye in the aqueous system; S_wat_ the molar solubility of the dye in water; and C_surf._ the molar concentration of the surfactant [8]. The solubilization power of a specific surfactant can thus be determined from the slope of its solubility curve after the CMC. If the molecular weights of the surfactant and the solubilisate are known, the SP can also be expressed as the weight of the compound solubilized per unit mass of surfactant.

If the average number of surfactant molecules in each micelle, the surfactant aggregation number; N_aggregation_ is not influenced by the solubilization of the dye, the solubilization capacity; Σ which is the average number of dye molecules solubilized in each micelle, can be defined as follows (Equation 4) [47]:

(4) $$\Sigma \;=\;N_{\mathrm{aggregation}}\;\times \;\mathrm{SP}$$

If one dye molecule is solubilized into each micelle, which may be the case for spherical micelles, the value of Σ becomes 1. The aggregation number can then be obtained from Equation 4 if the SP value is known. The N_aggregation_ value obtained is then often similar to that obtained by other methods [42,48]. However, the uncertainty is considerable since the micelles may grow into elongated or disc-like structures by the uptake of the dye and such large micelles may readily solubilize more than one dye molecule [49]. Characterization of surfactant micelles containing a water insoluble dye by light scattering techniques has indeed shown that the dye-containing micelles are often much larger than the micelles without dye [50]. For micelles of ionic surfactants this is particularly the case for systems with a high salt concentration. Electrolytes shield the charges of the surfactant head groups, which leads to an increase of the CPP and larger CPP values tend to give more elongated micelles. Some of the N_aggregation_ values based on the dye solubilization method that are given in the literature should therefore be reconsidered. Normally, the Σ values for spherical micelles are considerably less than unity, however.

The micelle-water partition coefficient (R_mic/wat_), which is the ratio of dye concentration in the micelle and in the surrounding water for a particular surfactant concentration can be written as:

(5) $$R_{\frac{\mathrm{mic}}{\mathrm{wat}}}\;=\;\frac{S_{\mathrm{total}}-S_{\mathrm{wat}}}{S_{\mathrm{wat}}}$$

This equation can be rewritten using Equation 3:

(6) $$R_{\frac{\mathrm{mic}}{\mathrm{wat}}}\;=\;\frac{\mathrm{SP}\times (C_{\mathrm{surf}.}-\mathrm{CMC})}{S_{\mathrm{wat}}}$$

The standard free energy of solubilization (ΔG_S_) can be then calculated from the following equation (Equation 7) [51]:

(7) $$\Delta G_{S}\;=\;R\;T\;\ln\left(R_{\mathrm{mic}/\mathrm{wat}}\right)$$

where R is the universal gas constant and T is the absolute temperature. It should be noted that in order to obtain thermodynamic data (e.g., ΔG_S_) correctly, the activity of the solute should be used instead of the concentration. The activity coefficient, which gives the deviations from ideality, reflects the interaction between solute and surfactant in the micelles. It may be determined by vapor pressure measurements [41].

The above mentioned values (*i.e.*, SP, Σ, R_mic/wat_, and ΔG_S_) are useful for quantitative determination of the solubilization efficiency of a surfactant and can be used for comparing different surfactants under various conditions (e.g., temperature, salt concentration, pH).

## 6. Important Factors in Dye Solubilization

### 6.1. Surfactant Concentration

**Figure 5:**
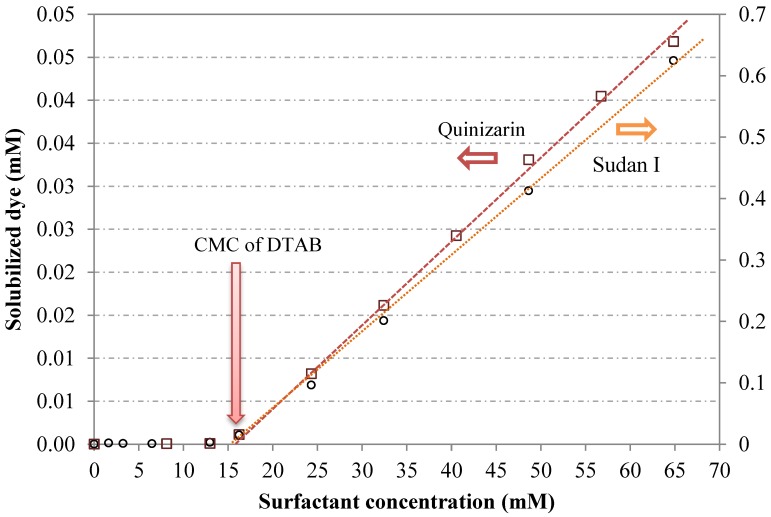
Solubilization of two hydrophobic dyes C.I. Solvent Yellow 14 (Sudan I) and C.I. Solvent Orange 86 (Quinizarin)in the presence of the cationic surfactant dodecyltrimethylammonium bromide (DTAB) at 21 °C. Data from [44,45].

The surfactant micelles are responsible for the solubilization of the dye and the onset of solubilization is at the CMC of the surfactant. Dye solubilization is, in fact, used as one of the methods for determination of the CMC [52]. The molar concentration of two hydrophobic dyes, C.I. Solvent Yellow 14 (Sudan I) and C.I. Solvent Orange 86 (Quinizarin), solubilized in an aqueous solution containing the cationic surfactant dodecyltrimethylammonium bromide (DTAB) is shown in Figure 5 as a function of surfactant concentration. The chemical structure of the dyes and the solubilization power for the dyes are provided in Table 2. As can be seen, the amount of dye solubilized at a surfactant concentration below the CMC is negligible and the onset of dye solubilization is 15.3 mM and 15.7 mM for Sudan I and Quinizarin, respectively [44]. These values are in good agreement with the CMC value of DTAB obtained from tensiometry (15.1 mM) and from conductometry (15.4 mM) [53]. It can also be seen that the dye solubilization increases almost linearly with increasing surfactant concentration beyond the CMC. For the majority of surfactants, this holds true for low to moderate surfactant concentration. When the concentration becomes so high that the viscosity goes up, which is usually an indication of rod-like micelles, there is no longer a linear relation between dye solubilization and surfactant concentration.

### 6.2. Surfactant Structure

As was discussed in Section 0, the solubilization power (SP) is an important parameter. Surfactants with different chemical structures give different SP values for a specific dye. This has led to a search for the optimal surfactant structure for solubilization of a certain dye. A few relevant examples of such studies are given below.

**Figure 6:**
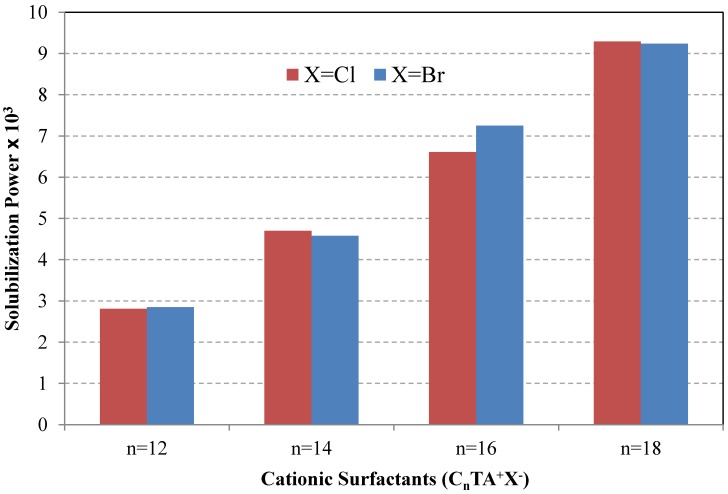
Solubilization of C.I. Solvent Red 25 (Sudan IV) in aqueous solutions of a series of cationic surfactants (C_n_TA^+^X^−^; n = 12,14,16,18 and X = Cl, Br) at 25 °C. Data from [54].

The solubilization properties of C.I. Solvent Red 25 (Sudan IV) in aqueous solutions of a series of alkyltrimethylammonium halides (C_n_TA^+^X^−^; n = 12,14,16,18 and X = Cl, Br) were investigated [54]. As can be seen from Figure 6, the molar solubilization power of these cationic surfactants in water increases almost linearly with an increase in number of carbon atoms in the alkyl chain (around 2.15 × 10^−3^ per two CH_2_ in the alkyl tail). This can be explained by an increase in the volume of the hydrophobic core of the micelle. However, the value of solubilization capacity (Σ) was found to be lower than unity, even for spherical micelles of cetyltrimethylammonium type (C_16_TA^+^X^−^; X = Cl, Br). The choice of counterion (chloride or bromide) was not important for the solubilization capacity [54].

**A** similar behavior was reported for solubilization of two other water insoluble dyes, C.I. Solvent Yellow 2 (Methyl Yellow) and C.I. Solvent Orange 2 (Orange OT), in aqueous solutions of a series of alkylpyridinium bromide (C_n_P^+^Br^−^; n = 10,12,14,16) [55,56], a series of alkyl sulfates (C_n_OSO_3_^−^Na^+^; n = 8,10,12,14) [47], and a series of fatty acid salts (C_n_OO^−^X^+^; n = 10,12,14,16,18 and X = Na, K), respectively. The solubilizing power increased linearly with increasing number of carbon atoms while the choice of counterion had a negligible effect [5,57].

Replacement of the CH_3_ terminal group in dodecyltrimethylammonium bromide (DTAB) with CF_3_ was found to have a negative effect on dye solubilization. This can be explained by the incompatibility of the CF_3_ group with the rest of the surfactant tail, as well as with the dye. The core of the micelle is no longer a good environment for the hydrophobic dye, which makes solubilization less energetically favorable. In addition, the micelle aggregation number is lower for the fluorinated than for the regular surfactant, which also works against solubilization. Taken together, the solubilization power for C.I. Solvent Orange 2 (Orange OT) is 30% lower in a micellar solution of the fluorine containing surfactant than in a micellar solution of regular DTAB [58].

**Figure 7:**
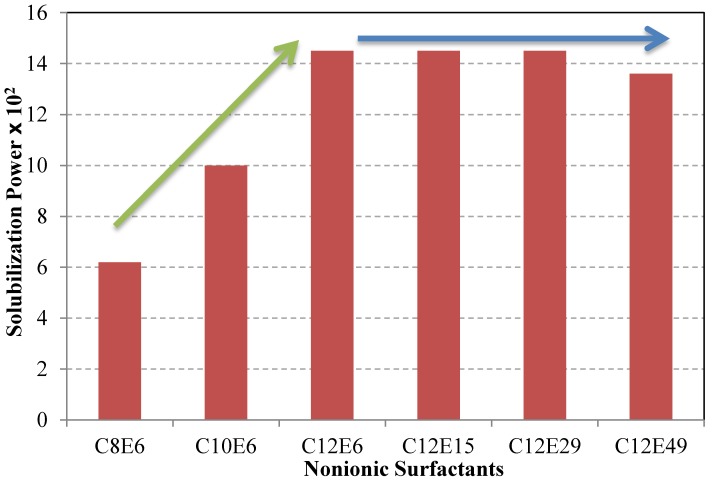
Solubilization of C.I. Solvent Yellow 6 (Yellow OB ) in aqueous solutions of a series of nonionic surfactants (C_n_E_m_; n = 8,10,12, m = 6,15,29,49) at 30 °C. Data from [59].

The solubilization of C.I. Solvent Yellow 6 (Yellow OB) was studied in the presence of a series of nonionic surfactants with various alkyl chain lengths and with different lengths of the polyoxyethylene chain. The nonionic surfactants are all fatty alcohol ethoxylates with the formula C_n_H_2n+1_(OCH_2_CH_2_)_m_OH, or C_n_E_m_ for short. As can be seen from Figure 7, the dye solubilization increased by increasing the length of alkyl tail (compare C_8_E_6_, C_10_E_6_, and C_12_E_6_), while it was almost unaffected by the length of polyoxyethylene chain (compare C_12_E_n_; n = 6,15,29,49) [59]. This clearly shows that the size of the hydrophobic core of the micelles is decisive for solubilization of a hydrophobic dye while the size of the outer region (see Figure 4) is not equally important. The CMC increases considerably when the polyoxyethylene chain is increased. This does not affect the SP value, however, because the solubilization power of a surfactant is defined as moles of solubilized dye per mole of micellized surfactant. If one would instead use mole of solubilized dye per gram of added surfactant, the efficiency would drop with the length of the polyoxyethylene chain because the more hydrophilic the surfactant, the larger is the fraction of non-micellized surfactant and the surfactant unimers do not contribute to the solubilization.

**Figure 8:**
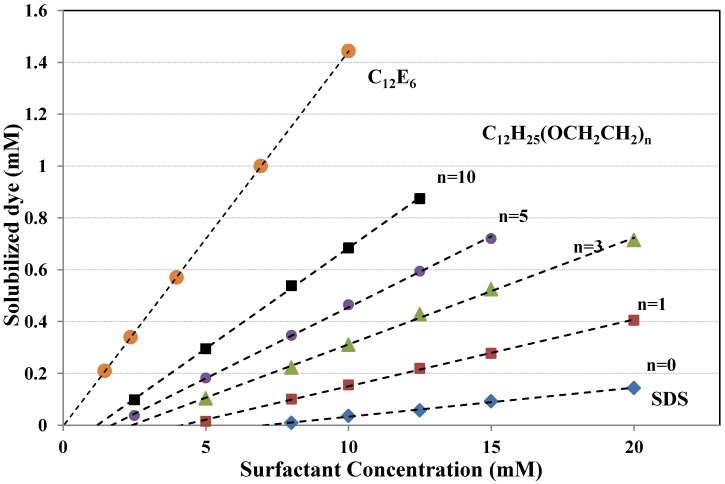
Amount of solubilized C.I. Solvent Yellow 6 (Yellow OB) as a function of the concentration of different surfactants. C_12_E_6_ is the nonionic surfactant and SDS stands for sodium dodecyl sulfate. SDS can also be written C_12_H_25_(OCH_2_CH_2_)_n_OSO_3_^−^Na^+^ with n = 0. The measurements were made at 25 °C. Data from [47,59].

In another extensive study, the solubilization of C.I. Solvent Yellow 6 (Yellow OB) was investigated in the presence of either an anionic surfactant with the structure C_12_(OCH_2_CH_2_)_n_OSO_3_^−^Na^+^; n = 0,1,3,5,10 or a nonionic surfactant, C_12_H_25_(OCH_2_CH_2_)_n_ OH, n = 7,10,13,15, and 20 (C_12_E_n_ for short). As can be seen, all surfactants have a dodecyl chain as hydrophobic tail. Again, the solubilization power of the nonionic surfactants was almost independent of the length of the polyoxyethylene chain (*i.e.*, the same trend as in Figure 7) and much higher than that of the anionic surfactants. The presence of oxyethylene groups in the structure of the anionic surfactants had a positive effect on the solubilization capacity. Figure 8 shows selected results from two similar studies [47,59]. It can be seen that as the surfactant goes from purely anionic (SDS), via the ether sulfates, C_12_(OCH_2_CH_2_)_n_OSO_3_^−^Na^+^, which become progressively more nonionic in character with increasing value of n (n = 0 is SDS), to the purely nonionic C_12_E_6,_ the solubilization increases. Note that Figure 8 does not show solubilization power; instead, it displays amount of solubilized dye as a function of added surfactant. One can then expect that the CMC value will be important because a surfactant with a low CMC will have a higher proportion of the added surfactant molecules in the form of micelles than a surfactant with high CMC and it is only the micelles that contribute to the solubilization. The trend shown in Figure 8 basically follows the CMC values with C_12_E_6_ having the lowest and SDS, *i.e.*, C_12_(OCH_2_CH_2_)_n_OSO_3_^−^Na^+^ with n = 0, the highest CMC. It is also conceivable that the difference in micelle shape may contribute to the difference in solubilization efficiency. While SDS and the other sulfates give spherical micelles at 25 °C, C_12_E_6_ give slightly elongated micelles [47,60]. 

The solubilization of two hydrophobic dyes, C.I. Solvent Yellow 14 (Sudan I) and C.I. Solvent Orange 86 (Quinizarin), in the presence of a series of surfactants was investigated. By comparing the efficiency of the two nonionic surfactants C_11_E_5_ and an alkylphenol ethoxylate, nona(ethylene glycol)monononylphenol ether, as well as two anionic surfactants, sodium dodecyl sulfate (SDS) and sodium dodecylbenzene sulfonate (SDBS), it was concluded that a surfactant with a straight alkyl tail gives better solubilization than a surfactant that contains an alkylaryl tail [45]. It was also found in the study that an aromatic ring or a double bond in the hydrophobic tail reduces the solubilization power, probably because they restrict the conformational freedom of the tails within the micelles. A high degree of flexibility of the chains that make up the interior of the micelle seems to be advantageous for solubilization of a hydrophobic dye [61].

The solubilization of several disperse dyes with various chemical structures were investigated at high temperature in the presence of 11 different dispersants and surfactants. The dispersing agents used were lignin sulfonates and condensation products of naphthalene sulfonic acid with formaldehyde. The surfactants investigated were different anionic and nonionic amphiphiles. It was concluded that the low molecular and more well-defined surfactants were more efficient as solubilizing agents than the higher molecular weight lignin sulfonates and naphthalene sulfonic acid-formaldehyde condensation products.

Gemini surfactants constitute a relatively novel type of amphiphile that exhibit an unusual strong tendency to self-assemble both in bulk and at interfaces. A gemini surfactant consists of two monomeric surfactants linked together at the head groups [62,63]. The solubilization power of cationic gemini surfactants has been thoroughly studied and it has been found that the solubilization efficiency of such a surfactant is often higher than that of the monomeric counterpart [43,44,64]. Much of the increased efficiency can be attributed to the fact that an ionic gemini surfactant typically has a CMC that is 1–2 orders of magnitude lower than the CMC of the corresponding monomeric surfactant [65,66,67,68]. Consequently, the solubilization starts at much lower surfactant concentration. The solubilization capacity varies with the type of dye, however, and a lower solubilization power for a gemini surfactant than for the corresponding regular surfactant has been reported for a small hydrophobic dye [43]. A comparison between gemini surfactants and regular surfactants in terms of solubilization capacity is complicated by the fact that their micelle shapes may differ.

**Figure 9:**
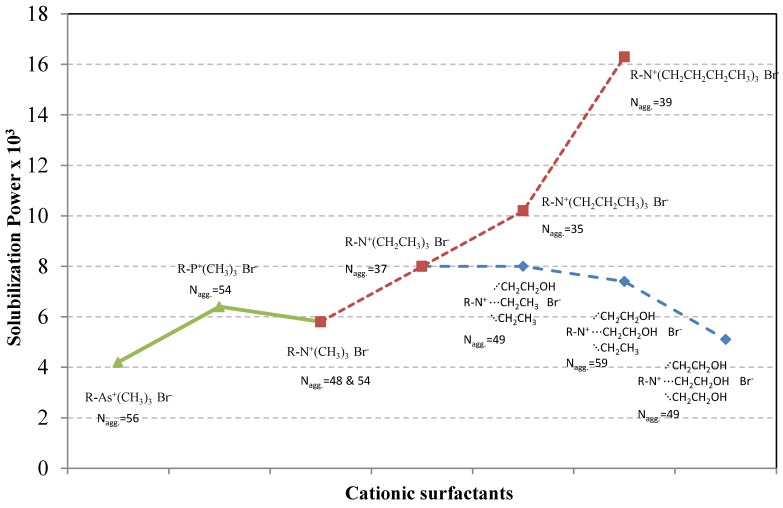
Effect of polar head group on molar solubilization power at 25 °C of the dye C.I. Solvent Orange 2 (Orange OT) by different cationic surfactants, all based on a decyl chain as hydrophobic tail and all having bromide as counterion. The aggregation numbers (N_agg_) are also given for each surfactant. Data from [69,70,71].

It is also important to understand the effect of the polar head group on dye solubilization. It has been suggested that the head group can affect the solubilization by at least two different mechanisms: (a) it may influence the aggregation number and thus the micelle size; and (b) the head group region (the outer region in Figure 4) can also serve as a site for solubilized dye molecules. This issue has been the topic of a number of studies [69,70,71]. The solubilization of C.I. Solvent Orange 2 (Orange OT) in aqueous solutions of a series of cationic surfactants, all based on decyl chains and having an ammonium head group with different substituents, was investigated. It was shown that when the polar head group can interact strongly with the dye, the solubilization can become very high. A larger and more hydrophobic head group increases the solubilization power, most likely because dye molecules can be solubilized not only in the inner part of the micelle but also in the outer region. The solubilization power of a number of cationic surfactants with different sizes of the polar head group is shown in Figure 9. The micelle aggregation numbers are also given. It can be seen from the figure that there is no correlation between N_aggregation_ and solubilization. Instead, the size of the alkyl substituents on the positively charged nitrogen seems to be a decisive factor. It can also be seen that replacing ethyl substituents by hydroxyethyl is detrimental to the solubilization power. Evidently, the increased polarity provided by the hydroxyl groups makes the outer region of the micelle an energetically less favorable environment for the hydrophobic dye.

Figure 9 also shows the effect of changing the charge-bearing atom in the cationic head of the surfactant on dye solubilization. Replacement of nitrogen by phosphorus does not much affect the solubilization power and replacement by arsenic leads to a decrease in solubilization. As can be seen from the figure, this seems not to be related to the micelle aggregation number. There is also no obvious connection to head group size, the trend of which is As^+^(CH_3_)_3_ > P^+^(CH_3_)_3_ > N^+^(CH_3_)_3_. One may speculate that the As^+^(CH_3_)_3_ head group gives a more polar environment than the other two.

There are a limited number of studies that deal with solubilization of hydrophobic dyes in micelles of polymeric surfactants. On a molar basis the solubilization power of these surfactants is much higher than that of conventional low molecular weight surfactants as their micelles are normally much bigger. On a weight basis, however, which is the commercially important measure, they are not very efficient as they have higher molecular weight [45]. Polymeric surfactants do have one advantage over conventional surfactants for dye solubilization, however. Since they self-assemble at extremely low concentrations, they are more resistant to dilution. All surfactant solutions lose their ability to solubilize hydrophobic molecules when they are diluted to below the CMC. Polymeric surfactants have very low CMC values and are therefore capable of retaining a solubilization efficiency down to very low surfactant concentration [72].

### 6.3. Dye Structure

There are several studies that show that the solubilization power (SP) depends not only on the surfactant but also on the chemical structure of the dye [4,5,43,44,45,57]. The values of solubilization power of the cationic surfactant dodecyltrimethylammonium bromide (DTAB) for a range of hydrophobic dyes with different structures are summarized in Table 2. Some observations can be made:

(a) Azo dyes, *i.e.*, dyes that contain the N = N bond, give higher SP values than the anthraquinone dyes (the last two dyes on the list). As can be seen from the table, this is not related to a difference in molecular weight. It is also not related to differences in polarity; C.I. Solvent Yellow 14 (Sudan I) and C.I. Solvent Orange 86 (Quinizarin) have approximately the same water solubility. Instead, the difference in the SP values can most likely be attributed to differences in conformational freedom of the dyes. Whereas anthraquinone derivatives are rigid, planar molecules, the azo dyes are relatively flexible because of low barrier for rotation around the C-N bonds [45].

(b) For azo dyes the SP value decreases with increasing molecular weight. This is the expected trend because it is more difficult for a micelle to accommodate a large than a small molecule.

(c) Polar substituents play a big role for the SP value and the more polar the better. Amino groups are particularly effective, as can be seen for both families of dyes. Polar substituents can provide two effects: (1) they will increase the water solubility; and (2) they will make the dye dissolve not only in the most hydrophobic part of the micelle but also in the less hydrophobic palisade region, *i.e.*, the region just below the head group region (see Figure 4) [73]. Both effects will raise the SP value.

**Table 2:** Solubilization power of dodecyltrimethylammonium bromide (DTAB) for different water insoluble dyes at 23–25 °C.

Dye	Dye molecular weight (g/mol)	Solubilization power (10^3^ mol/mol)	Reference
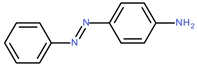 C.I. Solvent Yellow 1	197.2	256	[43]
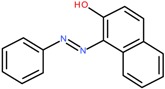 Sudan I/C.I. Solvent Yellow 14	248.3	13.5	[44,45]
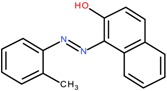 Orange OT/C.I. Solvent Orange 2	262.3	11.5	[74]
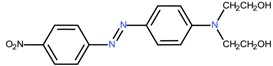 C.I. Disperse Red 19	330.3	2.7	[43]
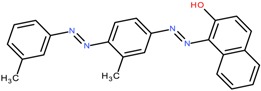 Sudan IV / C.I. Solvent Red 25	380.4	2.2 and 2.8	[54,75,76]
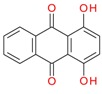 Quinizarin / C.I. Solvent Orange 86	240.2	0.9	[44,45]
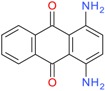 C.I. Disperse Violet 1	238.2	4	[43]

### 6.4. Temperature

**Figure 10:**
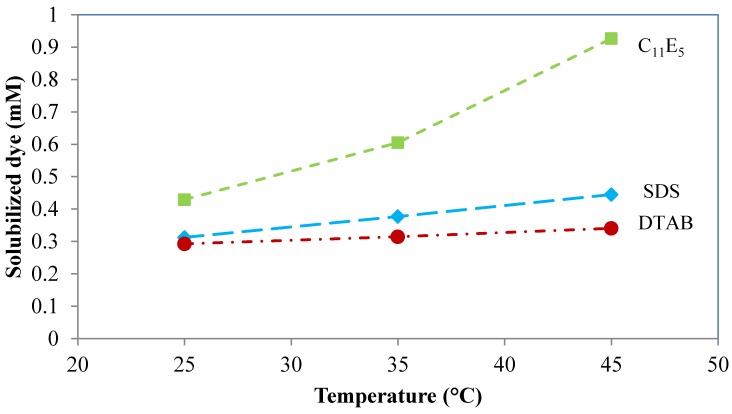
Amount of solubilized dye C.I. Solvent Yellow 14 (Sudan I) as a function of temperature for three different surfactants, the anionic sodium dodecyl sulfate (SDS), the cationic dodecyltrimethylammonium bromide (DTAB) and the nonionic penta(ethylene glycol)monoundecyl ether (C_11_E_5_) at a fixed concentration of 10 g/L [44].

The amount of solubilized dye is affected by the temperature and the effect depends both on the structure of the dye and on the type of surfactant used in the formulation. Nonionic surfactants are particularly temperature sensitive, as is illustrated by Figure 10. Three different surfactants, the anionic sodium dodecyl sulfate (SDS), the cationic dodecyltrimethylammonium bromide (DTAB) and the nonionic penta(ethylene glycol)monoundecyl ether (C_11_E_5_) were used for solubilization of the hydrophobic dye Sudan I [45]. A similar trend, *i.e.*, a small increase in solubilization with an anionic and a cationic surfactant and a large increase with a nonionic surfactant, has been reported for other dyes [5,64]. The moderate increase of the solubilized amount with an increase in temperature seen for the anionic and the cationic surfactant is a result of increased thermal agitation and larger available space in the micelles [77]. This effect also contributes to the very pronounced influence of temperature seen with the nonionic surfactant but the major contribution in this case is that the polyoxyethylene chain of the nonionic surfactant gradually loses hydration water as the temperature goes up. This leads to an increase in the critical packing parameter of the surfactant (see Section 4), which, in turn, causes a transition from relatively spherical to elongated micelles [8,78]. These larger micelles can more readily solubilize hydrophobic molecules [8,78].

### 6.5. Addition of Salt

**Figure 11:**
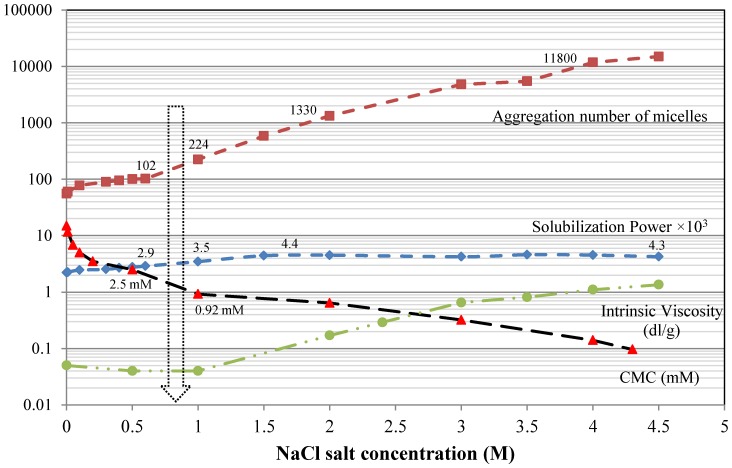
Aggregation number, solubilization power, intrinsic viscosity and critical micelle concentration (CMC) of dodecyltrimethylammonium chloride as a function of NaCl concentration. The dye used is C.I. Solvent Red 25 (Sudan IV) and the temperature was 25 °C. Data from [76,79,80].

It is known from many reports that addition of salt increases the solubilization of a water insoluble dye in a solution of an ionic surfactant [6,45,47,54,57,76]. An example of the effect of NaCl addition on the solubilization power of dodecyltrimethylammonium chloride (DTAC) towards C.I. Solvent Red 25 (Sudan IV) is shown in Figure 11. The aggregation numbers, the intrinsic viscosity and the CMC values of DTAC at the various salt concentrations are also provided in the figure [76,79,80]. As can be seen, the solubilization power and the aggregation number increase while the CMC decreases when the salt concentration is increased. Electrolytes in a solution of an ionic surfactant will shield the charges of the polar head group. This gives a more hydrophobic surfactant that self-assembles at lower concentration. It also leads to an increase in the critical packing parameter (see Section 0), which causes a transition from more spherical to more elongated micelles (oblates or prolates) [76,79,80]. This change of the shape of the micelles makes the aggregation number increase. The viscosity increase is mainly caused by the successive transition from spherical to elongated micelles. Judging from both the viscosity curve and the aggregation number curve the transition from spherical to elongated micelles starts around a NaCl concentration of 1 M.

As can be seen from Figure 11, the solubilization increases with increasing salt concentration but the effect is not dramatic. This confirms the general picture that as the micelles grow larger, their ability to solubilize hydrophobic substances increases. One may compare the results from Figure 10 and Figure 11 in this respect. Micelles of nonionic surfactants grow into elongated structures by an increase in temperature and, as seen from Figure 10, the amount of solubilized dye at a fixed surfactant concentration increases by a factor of 2 when the temperature is raised from 25 °C to 45 °C. Micelles of ionic surfactants are not much influenced by temperature variations but are instead much affected by electrolyte addition. The solubilization power of the cationic surfactant of Figure 11 increases by the same factor of approximately 2 when going from distilled water to a NaCl concentration of 1.5 M.

It is also seen in Figure 11 that the solubilization power remains constant above a NaCl concentration of around 1.5 M in spite of the fact that the aggregation number increases rapidly. This clearly shows that once the micelles have grown to a certain size the solubilization is not facilitated by the micelles becoming even more extended, ultimately often reaching a worm-like or thread-like structure. Obviously the same amount of dye is solubilized in a smaller amount of worm-like micelles as in a larger amount of only slightly elongated micelles (assuming the same total amount of surfactant) [76].

### 6.6. Addition of Polyelectrolytes

Mixtures of surfactants and polymers are common in many formulations. Surfactants and polymers in aqueous solution may interact by hydrophobic interactions, which are always attractive, and by electrostatic interactions, which may be either attractive or repulsive. These interactions are influenced by parameters such as pH, electrolyte concentration and temperature and the topic has been treated in several review papers and book chapters [9,81,82,83]. In the majority of the cases, addition of a water soluble polymer (usually a polyelectrolyte or a surface active nonionic polymer) to an aqueous surfactant solution shifts the onset of dye solubilization to lower surfactant concentration. This is due to formation of mixed assemblies (sometimes called polymer induced micellization) at a concentration considerably lower than the CMC of the surfactant. The surfactant concentration at which this occurs is called the critical association concentration (CAC) and a water insoluble dye can be used as a probe for determination of the CAC of such systems [84,85,86,87].

**Figure 12:**
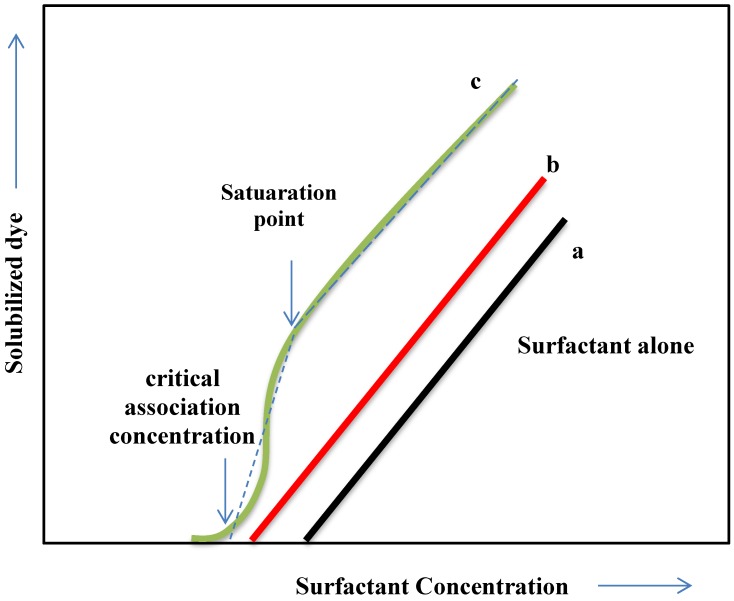
Solubilization of a water insoluble dye by surfactant-polyelectrolyte mixtures can take different paths. See text for explanation of the curves.

Published results from a number of solubilization experiments with surfactant-polyelectrolyte systems are summarized in Figure 12. The curves represent different scenarios, all with varying concentration of surfactant and with constant concentration of polyelectrolyte.

Curve (a) represents the situation for surfactant alone but also for the case when there is no interaction at all between the surfactant and the polyelectrolyte. The size and shape of the micelles are the same with and without the polyelectrolyte present. This may be the case if the polyelectrolyte carries the same charge as the surfactant head group [88,89].

In Curve (b) the polyelectrolyte-surfactant mixture has approximately the same solubilization power as the surfactant alone. The curve is linear but slightly moved towards lower surfactant concentration. This represents the case where normal surfactant micelles are responsible for the solubilization of the dye but the presence of the polyelectrolyte induces a shift of the CMC towards lower concentration [90].

Curve (c) is the most interesting case. The presence of the polyelectrolyte induces self-assembly at very low surfactant concentration. This is an example of a CAC, mentioned above. The very rapid and powerful increase in solubilized dye after an initial induction period is a sign of a cooperative self-assembly involving the polymer, as well as the surfactant. Both the polymer and the surfactant contribute to the solubilization. This is, for instance, the case when the polyelectrolyte has hydrophobic substituents that can form mixed micelles with the surfactants. At some point the curve levels out and becomes linear with the same slope as for Curves (a) and (b). At that point, called the “saturation point” on Figure 12, the polyelectrolyte has become “saturated” with surfactant. Beyond that point the self-assembly structures that form are regular surfactant micelles. Increasing the polymer concentration would be a way to move the “saturation point” further up and to the right [91].

Not only polyelectrolyte-surfactant systems can give Curve (c)-type of slopes, but also combinations of a surfactant with a hydrophobically modified nonionic water soluble polymer can exhibit this behavior. Combinations of sodium dodecyl sulfate with hydrophobically modified ethylhydroxyethylcellulose are relevant examples [92,93].

Combinations of polymers and surfactants are not always favorable, however. For example, adding a polyelectrolyte to a solution of an oppositely charged surfactant may lead reduced solubilization power and even to precipitation.

### 6.7. Influence of pH

**Figure 13:**
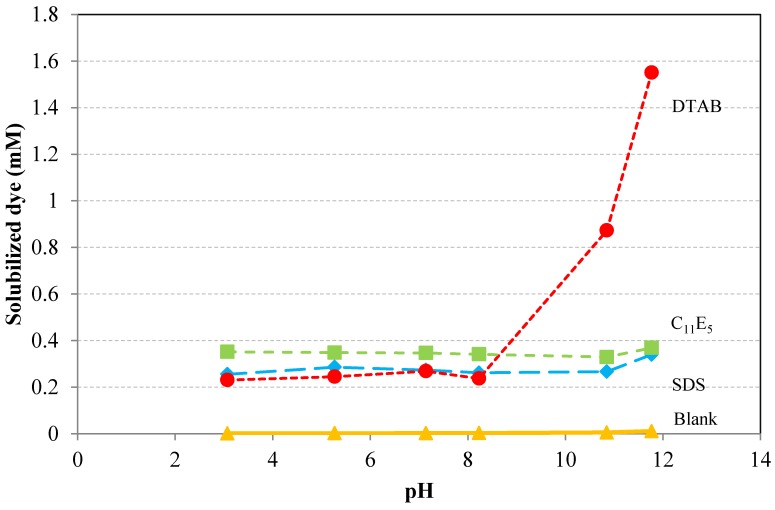
Effect of pH on solubilization of C.I. Solvent Yellow 14 (Sudan I) at 21 °C for dodecyltrimethylammonium bromide (DTAB), sodium dodecyl sulfate (SDS), and penta(ethylene glycol)monoundecyl ether (C_11_E_5_), all at a concentration of 10 g/L [45].

There are only few papers dealing with the pH effect on dye solubilization in surfactant solutions. A reason for the lack of studies of this topic may be that the pH effect is believed to be negligible. Figure 13 shows that this is not always the case, however. The figure shows the effect of pH on solubilization of the dye Sudan I in the presence of dodecyltrimethylammonium bromide (DTAB), sodium dodecyl sulfate (SDS), and penta(ethylene glycol)monoundecyl ether (C_11_E_5_). The solubilization of the dye increases rapidly at a pH greater than 8 for the cationic surfactant, while the solubilization of the same dye is almost unaffected by pH variations for the anionic and nonionic surfactants. It should be noted that: (a) the wavelength of maximum absorption (λ_max_) of the dye remains constant at 485-486 nm within the pH interval and there is not any blue or red shift in the UV-Vis spectra; (b) the dye solubilization in absence of any surfactant is negligible in the pH interval 3–12; and (c) the amount of added NaOH to increase the pH of the solution is not high enough to affect the CMC or to induce growth of the micelles, as is evident from the almost constant solubilization values for SDS and C_11_E_5_. Most likely, the dramatic increase in solubilization of Sudan I by the cationic surfactant DTAB at high pH is due to deprotonation of the phenolic hydroxyl group of the dye. (The structure of Sudan I is given in Table 2). This deprotonation is driven by the attractive interaction between the ionized dye and the cationic surfactant. One may speculate that non-ionized dye molecules will be solubilized in the hydrophobic, inner part of the micelle while ionized dye molecules will be incorporated in the upper palisade layer and in the outer layer of the micelle. Another way to view the situation is that the deprotonated dye will act as an anionic surfactant, forming mixed micelles with the cationic DTAB. Formation of such mixed micelles are thermodynamically very favorable and the micelles can be elongated and large [45]. Surfactant induced deprotonation will not take place with anionic or nonionic surfactants, of course. One may therefore assume that the level of solubilized dye of around 0.3 mM represents solubilization in the inner, hydrophobic part of the micelle for all three surfactants.

## 7. Location of Solubilized Dye in the Micelle

There is a polarity gradient from the very hydrophobic core of the micelle towards the hydrophilic head group region (Figure 4) Fluorescence techniques can be used for determination of the polarity [94,95,96]. This is key for understanding the location of a dye molecule within a micelle.

In the majority of the papers discussed in this review that concern solubilization of a water-insoluble dye in surfactant micelles, it is assumed that the dye is located in the palisade region of the micelles [11,12,13,43,44,45,64].

**Figure 14:**
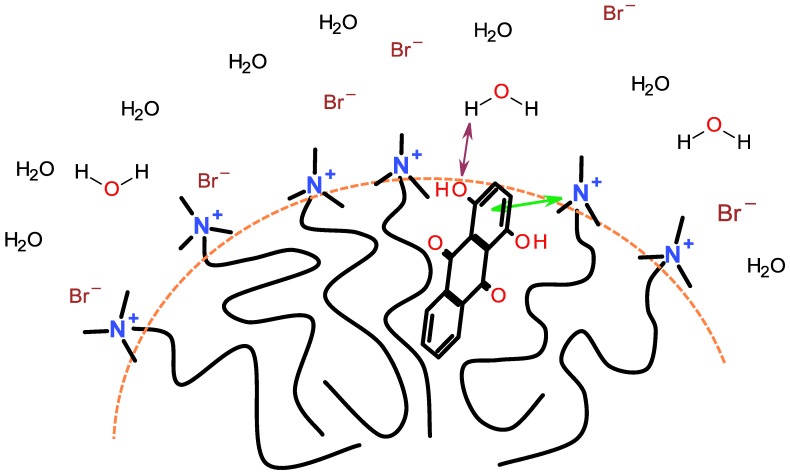
Assumed location of the dye C.I. Solvent Orange 86 (Quinizarin) in a micelle of a cationic surfactant.

Many water insoluble dyes have OH or NH_2_ substituents (see Table 2), and such dye molecules have been shown to reside just below the outer region of the micelle. It is likely that in many instances the dye will adapt an orientatione such that a polar substituent of the dye will interact with the surfactant head group by a combination of intermolecular interactions. The rest of the dye molecule will then protrude down into the micelle. Figure 14 shows one example of an assumed orientation of a dye, Quinizarin in a micelle of a cationic surfactant. The figure shows an attractive π-cation interaction between one of the aromatic rings of the dye and a quaternary ammonium group of the surfactant.

In special cases, such as for the pair Sudan I-DTAB, discussed in the previous section, the dye may be ionized at high pH, which can lead to a very strong electrostatic interaction with the polar head group of the surfactant.

**Figure 15:**
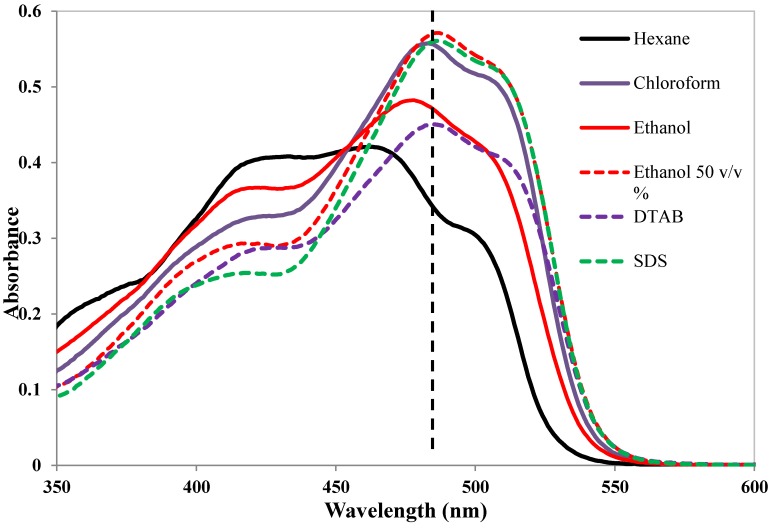
Absorption spectra of the dye C.I. Solvent Yellow 14 (Sudan I) in micellar solutions of DTAB and SDS and in different solvents. “Ethanol 50 v/v %” stands for equal volumes of water and ethanol.

The position of the dye can be determined indirectly by comparing the UV-Vis spectrum of the dye in surfactant solution with its adsorption spectra in solvents with different polarity. If the absorption spectrum of a dye solubilized in surfactant micelles is similar to that in a polar solvent such as ethanol but different from that in a non-polar solvent such as n-hexane, one may assume that the dye is situated in the moderately polar palisade region. If, on the other hand, the absorption spectrum of the dye solubilized in the surfactant solution resembles the spectrum in n-hexane, the dye is most likely situated in the core region. [43,44,47,97,98,99]. Figure 15 shows the principle. The absorption spectrum of the dye Sudan I in aqueous solutions of DTAB and SDS is compared with the spectra in solvents with different polarity. As can be seen, the spectra for both surfactant solutions are very similar to that for ethanol-water 50:50.

Nonionic surfactants differ from ionic surfactants in that their polar head group region is very large. The polyoxyethylene chains may be much longer than the hydrophobic tail. This thick shell of polyoxyethylene chains that cover the micelle core is hydrated and it has been shown by ^17^O spin relaxation NMR that each oxyethylene unit binds around 3 water molecules and that the bound water is relatively equally distributed through the head group layer; thus, there is no strong polarity gradient in the shell [100]. The outer region of such micelles is polar but the polarity is still considerably lower than the polarity of the outer region of ionic surfactant micelles, sometimes referred to as the Stern region. This means that dye molecules with polar substituents may be incorporated also in the outer region of nonionic surfactant micelles to a larger extent than for ionic surfactant micelles. Since the solubilization power is approximately the same regardless of the length of the polyoxyethylene chain (see Figure 7) it is likely that the dye accumulates in the innermost part of the outer region The possibility for the dye to be incorporated also in the hydrophilic shell is probably the reason why nonionic surfactants often have higher solubilization power than ionic surfactants [47,101].

## 8. Solubilization in Mixed Micelles

Surfactant mixtures are common in many applications and the use of a combination of surfactants instead of a single one often results in improved performance. Mixtures of a nonionic surfactant with an ionic surfactant are particularly common, for instance for cleaning applications, and such mixtures may exhibit synergism with respect to CMC. This means that the CMC of the mixture is lower than the CMCs of the individual surfactants [8,9]. Since low CMC values are generally beneficial for solubilization of hydrophobic substances, such as dyes, one may anticipate that such mixtures would be commonly used in dyeing formulations.

It turns out, however, that in the majority of cases a mixture of an ionic surfactant and a nonionic surfactant does not give better solubilization of a hydrophobic dye than the use of one surfactant only [45,101]. An explanation to why addition of an ionic surfactant to a nonionic surfactant based system is not advantageous is that the character of the outer layer of the micelle is changed in an unfavorable way. As discussed in the previous chapter, one reason for the high solubilization power of nonionic surfactants is that dye molecules with polar substituents may be incorporated also in the outer region of nonionic surfactant micelles. This is not the case—at least not to the same extent—for ionic surfactant micelles. Mixing in an ionic surfactant means that the character of the polar shell that surrounds the micelle core will be changed in a way that is negative with respect to solubilization of dye molecules in this region. The previously uncharged layer will now contain charges and become considerably more polar, which will make it a less suitable environment for the dye.

## 9. Conclusions

This review has had a focus on solubilization of hydrophobic dyes in surfactant solutions. The solubilization power is related to the structure of both the surfactant and the dye. Dye molecules are mainly incorporated in the palisade region of the surfactant micelles but sometimes the core region can be the location and sometimes the dye can also be situated in the outer region, where the surfactant head groups are. The majority of dyes have polar substituents, such as hydroxyl and amino groups, and these can interact with surfactant polar head groups by a combination of intermolecular interactions. Such interactions are beneficial for the solubilization efficiency. In some cases, particularly with cationic surfactants, the polar head group can induce ionization of the dye and the ionic dye can then interact favorably with the oppositely charged surfactant.

The effect of adding salt, varying pH and temperature, and introducing polyelectrolytes into the system has been discussed from a surface chemistry viewpoint and with many references to the scientific literature.
